# Allogeneic hematopoietic stem cell transplantation to cure sickle cell disease: A review

**DOI:** 10.3389/fmed.2023.1036939

**Published:** 2023-02-23

**Authors:** Nishka Bhalla, Anjali Bhargav, Sandeep Kumar Yadav, Aloukick Kumar Singh

**Affiliations:** ^1^Centre for Stem Cell Research, Christian Medical College, Vellore, Tamilnadu, India; ^2^University of Texas MD Anderson Cancer Center, Houston, TX, United States

**Keywords:** sickle cell disease, hematopoietic stem cell transplantation, reduced intensity conditioning, veto cell, bone marrow transplantation

## Abstract

Sickle cell disease (SCD) had first been mentioned in the literature a century ago. Advancement in the molecular basis of the pathophysiology of the disease opens the door for various therapeutic options. Though life-extending treatments are available for treating patients with SCD, allogeneic hematopoietic stem cell transplantation (HSCT) is the only option as of yet. A major obstacle before HSCT to cure patients with SCD is the availability of donors. Matched sibling donors are available only for a small percentage of patients. To expand the donor pool, different contrasting approaches of allogeneic HSCT like T-cell replete and deplete have been tested. None of those tested approaches have been without the risk of GvHD and graft rejection. Other limitations such as transplantation-related infections and organ dysfunction caused by the harsh conditioning regimen need to be addressed on a priority basis. In this review, we will discuss available allogeneic HSCT approaches to cure SCD, as well as recent advancements to make the approach safer. The center of interest is using megadose T-cell-depleted bone marrow in conjugation with donor-derived CD8 veto T cells to achieve engraftment and tolerance across MHC barriers, under reduced intensity conditioning (RIC). This approach is in phase I/II clinical trial at the MD Anderson Cancer Centre and is open to patients with hemoglobinopathies.

## 1. Introduction

Sickle cell disease (SCD) is an autosomal recessive inherited genetic disorder caused by a single-nucleotide mutation in the β-globin gene, which leads to the substitution of glutamic acid by valine at position 6. Hemoglobin with this mutation is referred to as hemoglobin S (HbS). HbS polymerizes to form fibers, which change the shape of RBCs into a sickle shape, causing blockage of the circulation and resulting in a sickle cell crisis, hence the name sickle cell disease. HbS polymerization also leads to intravascular hemolysis of red blood cells (RBCs) and subsequent release of hemoglobin and other components into the plasma. This abnormality in hemoglobin may trigger several acute and chronic clinical manifestations such as vaso-occlusive crisis, splenic sequestration crisis, acute chest syndrome, and stroke (1–5). The disease was first described by Dr. James Herrick in 1910 in a 20-year-old patient suffering from severe anemia and malaria (6). During the patient’s blood smear examination, Dr. Herrick observed unusually shaped red blood cells that led him to conclude that the possible reason for the disease status is some unknown changes in red blood corpuscles. In 1949, the pioneering work by Linus Pauling showed that sickle cell disease is caused by abnormal hemoglobin (7).

Sickle cell disease is quite prevalent worldwide with the diagnosis of 300,000 new cases every year, and this number could rise to 400,000 by 2050 (8, 9). In the United States, one among 600 African Americans have been reported having SCD, and the disease affects 100,000 Americans (10–12). In sub-Saharan Africa, the mortality rate among newborns to 5-year-olds is 75% (13). However, the picture of the life expectancy of individuals born with SCD in the developed world is quite different, and the majority of SCD-affected subjects attain adulthood age. Nevertheless, the average life expectancy in well-resourced countries is much lower than that of a healthy individual (14–18). The aforementioned prevalence figures describe the magnitude of the problem worldwide; however, SCD has been recognized as a global public health problem by the WHO as early as 2006.

In the last few decades, tremendous advancement has been gained in understanding the pathogenesis of SCD; however, attempts toward the development of a treatment strategy were comparatively slow. Currently, allogeneic hematopoietic stem cell transplantation is the treatment of choice for patients with severe SCD. The HSCT is successful in treating patients with SCD when there is the availability of human leukocyte antigen (HLA)-matched sibling donors; however, its utility is extremely limited in HSCT across MHC barriers. Thus, the scarcity of suitable donors is an existing problem with HSCT. Allogeneic HSCT (allo-HSCT) often has to be used in such cases where a patient is in need of HSCT and the suitably matched donor is lacking. However, this approach is found to be attributed to graft failure after allo-HSCT and the occurrence of GvHD (19–22). The occurrence of GvHD is not the only limitation associated with allo-HSCT, it may be associated with conditioning-related toxicity and graft rejection. Moreover, new therapeutic approaches have been invented recently for the treatment of SCD and other hemoglobinopathies as well. The invention of new diagnostic tools like next-generation sequencing (NGS) together with gene therapy as a therapeutic option revolutionized the treatment options for hemoglobinopathies including SCD (23, 24). The NGS allowed the identification of specific genetic alternation responsible for hemoglobinopathy and gene therapy enables the treatment of genetic disorders through the transplantation of gene-corrected autologous HSCT (25). The autologous gene-corrected hematopoietic stem cells can home to their niches and start hematopoiesis without the risk of GvHD and rejection. Therefore, these corrected hematopoietic stem cells can maintain themselves for a lifetime and leads to the production of corrected RBCs and other cells as well. Gene therapy mainly involves two approaches: lentiviral vector correction and gene editing. In this review, we will mainly focus on the recent advancements with safer allogeneic HSCT approaches to cure SCD.

## 2. Historical perspective of SCD

**Figure 1:**
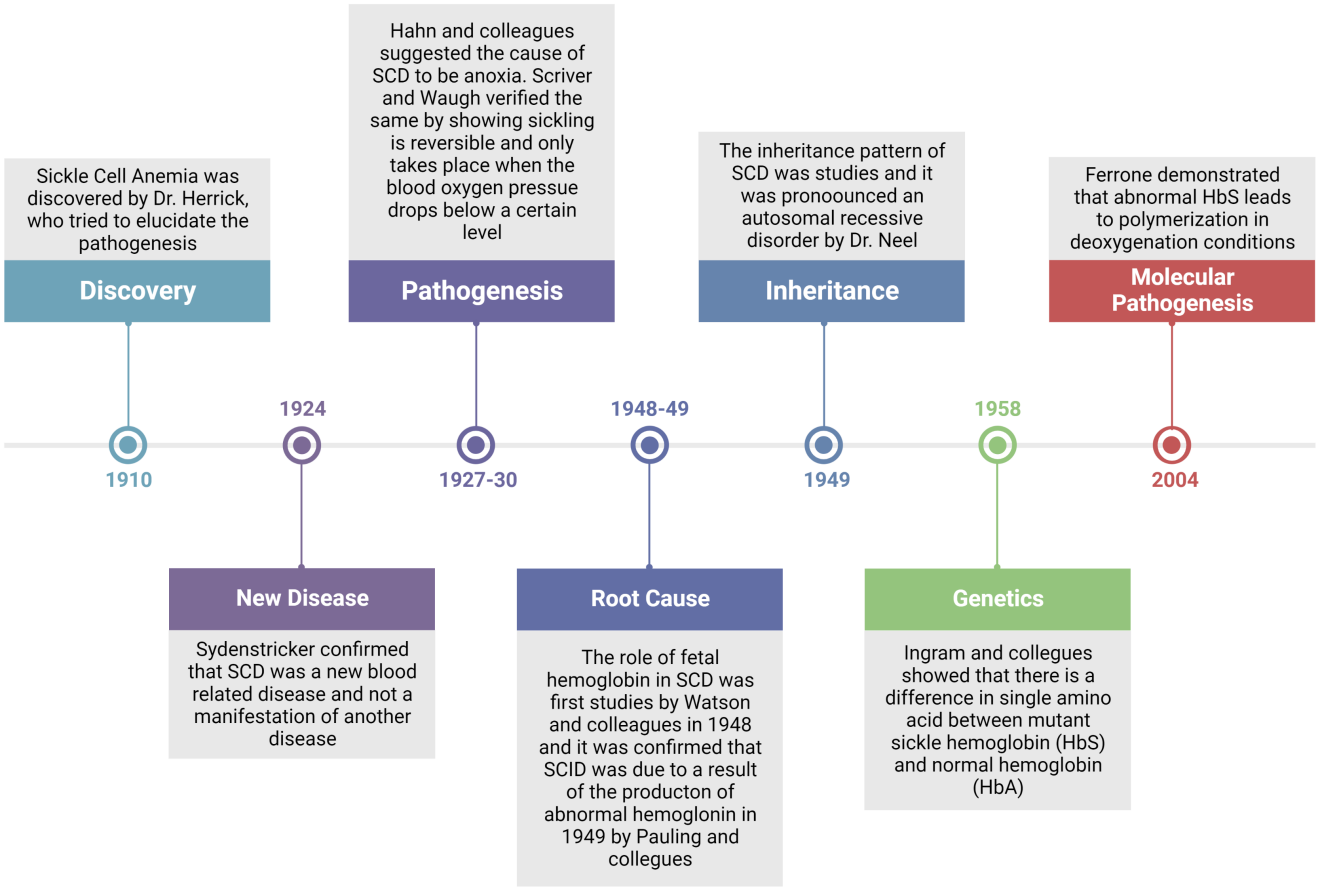
Timeline review of major historical events since the discovery of the disease and up to the established molecular pathogenesis of the disease.

Since the discovery of SCD in 1910 by Dr. Herrick, a lot of efforts have been made to elucidate a clear picture of the pathogenesis of the disease (Figure 1). Of noteworthy that with the given symptoms, he was not sure that this was a blood-related disorder or manifestation of another disease. However, up to now, several cases of SCD were identified and described, supporting the belief that it was completely a new disease (26). Three years later in 1927, Hahn and colleagues suggested that anoxia is the principal cause of RBC sickling by demonstrating that shape changes could be induced by saturating a cell suspension with carbon dioxide (27). This concept was again verified by Scriver and Waugh in a case report where they showed that the number of sickle cells in the blood could be changed by making partial changes in oxygen pressure; that is a reversible reaction, and that sickling takes place only when the oxygen pressure falls below pressure of 45 mm Hg (28). Furthermore, in 1948, Watson and colleagues reported the importance of fetal hemoglobin (HbF) in the extension of the sickling period among newborns when compared with mothers with sicklemia (29). These pioneering reports led Linus Pauling to hypothesize that disease might originate from abnormal hemoglobulin and he validated the hypothesis in 1949 by differences in the migration pattern of sickle and normal hemoglobin by gel electrophoresis (7). In the same year, the autosomal recessive inheritance pattern of the disease was elucidated by Dr. Neel (30). Thereupon in 1958, Ingram and colleagues for the first time provided the genetic evidence of the disease that there is a difference in single amino acid between mutant sickle hemoglobin and normal hemoglobin (HbA) (31). Thereafter, it could be demonstrated in 2004 that abnormal HbS leads to polymerization in deoxygenation conditions (32). These studies provided the platform for further investigation of SCD on a molecular basis.

## 3. Pathophysiology of SCD

The term SCD comprises a group of disorders among which disease pathology results due to the inheritance of the mutated sickle cell gene either homozygously or as a double heterozygote in combination with another gene. Thus, the disease pathology is mainly influenced by hemoglobin genes individually. However, among the majority of the population, the most frequent genotype manifesting disease is homozygous sickle cell (33). Over the 12 decades, four major pathological processes, namely HbS polymerization, vaso-occlusion, hemolysis-mediated endothelial dysfunction, and sterile inflammation, have emerged and are characterized (34).

**Figure 2:**
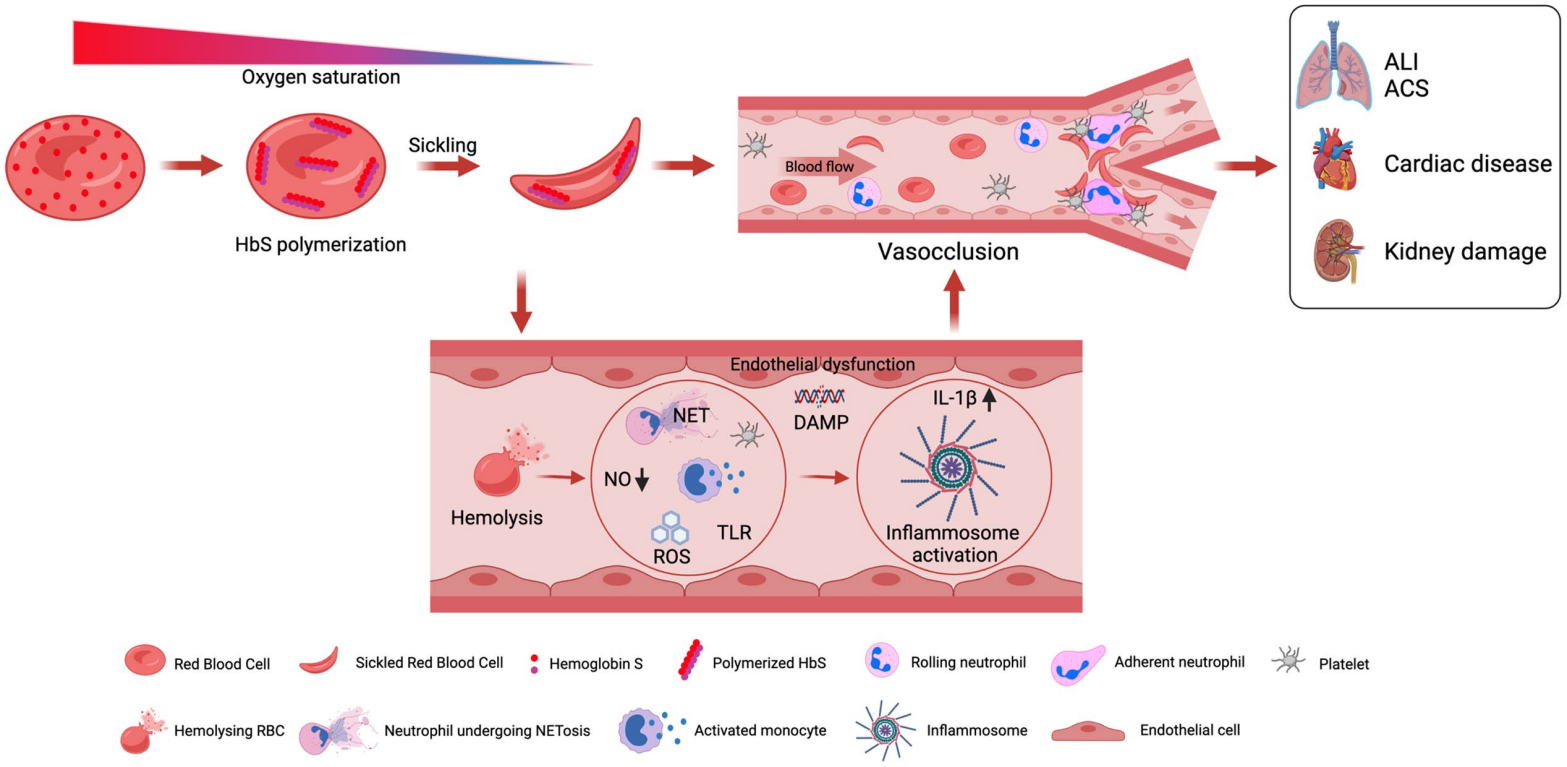
A graphical illustration of pathophysiologies associated with sickle cell disease.

Hemoglobin S polymerization under low oxygen is the primary event that leads to the complex pathophysiology and severe manifestations of SCD (Figure 2). Under the deoxygenated condition, the change of glutamic acid with valine triggers interactions with other hemoglobin molecules that cause aggregation of hemoglobin molecules into the large polymers. The polymerization of deoxygenated HbS causes structural and functional changes in RBCs. These distorted or damaged rigid sickle-shaped RBCs lost their flexibility and become more adhesive in nature. The process of sickling is not a continuous process; the sickling and unsickling occur at regular intervals and one of the major effects of the sickling and unsickling cycle is that it decreases the life span of RBCs (35, 36). The clinical outcome is an acute systemic painful vaso-occlusive crisis (VOC) due to the occlusion of blood vessels in every compartment of the body and chronic hemolytic anemia (37). The periodic recurrence of painful VOC is an essential feature of SCD.

All these events lead to the induction of a pro-inflammatory response that involves neutrophils, platelets, and vascular endothelium (34). It has been well established that nitric oxide (NO) is essential for normal vascular function (Figure 2). However, in SCD, there is a continuous release of hemoglobin from distorted cells from hemolysis depletes hemopexin and haptoglobin, a consequence of which is the reduction in the bio-availability of nitric oxide (NO), and vascular endothelial dysfunction that causes organ damage in SCD (38). The NO prevents endothelial damage by inhibiting, neutrophils, platelets, and the expression of adhesion molecules like VCAM-1 and ICAM-1 at the transcriptional level (39). Among patients with SCD, the levels of neutrophils, monocytes, and platelets remain above that of normal healthy subjects, which further get more increased during acute events (40).

To this end, various studies reported the correlation between neutrophilia and the severity of SCD (41, 42). A recent study reported that neutrophil interactions with RBCs and endothelium play a central role in VOC by increasing the expression of E and P selectins identified as current therapeutic targets to deal with VOC (43). In addition, the *in vivo* studies in SCD mice also showed that the interaction of neutrophils with adherent leukocytes can induce VOC (44). There are a series of reports that suggests the potential role of neutrophilia in the severity of SCD by triggering alternation in neutrophil surface marker expression, adhesion, migration, and intracellular oxidative stress (42). However, the underlying mechanism remains elusive. Furthermore, vitamin E levels remain diminished in patients with SCD, and neutrophils acquire a pro-inflammatory phenotype due to the loss of antioxidants. Furthermore, the administration of granulocyte-macrophage colony-stimulating factor (GM-CSF) and granulocyte colony-stimulating factor (G-CSF) in order to correct neutropenia worsens the disease status, suggesting the role of neutrophils in SCD pathogenesis (45). Thus, it was evident from various studies that anomalous interactions between endothelial cells, platelets, erythrocytes, and leukocytes comprise the pathophysiology of SCD during acute pain crises.

## 4. Therapeutic strategies for the treatment of SCD

The discovery of these pathophysiological targets has led the insights for the initiation of clinical trials by targeting platelets, adhesion molecules, and coagulation for the prevention of VOC-induced pain in SCD (46–49). To this end, in the transgenic SCD model, it has been demonstrated that anti-P-selectin can efficiently inhibit the adhesion of both sickle RBC and leukocytes to endothelial cells, suggesting a critical for P-selectin in cell adhesion (50). Very recently a humanized monoclonal antibody crizanlizumab (Adakveo) has been reported to block the P-selectin interaction with circulating leukocytes and the therapy was observed to be significantly effective in lowering the rate of sickle-related pain crises. This study also showed that the reduction in the annual crisis was more significant in patients those patients (50%) who were treated only with crizanlizumab (5 mg/kg) than those who received both the crizanlizumab and hydroxyurea combination therapy (32.1%) (51). Subsequently, this drug was got approved by the FDA in 2019. A study conducted in the SCD Berkeley mice model reported that a synthetic pan-selectin inhibitor, rivipansel (GMI-1070), can effectively inhibit E-selectin-mediated adhesion as well as RBC-leukocyte interactions, leading to improved microcirculatory blood flow and improved survival (52). Very recently, the clinical trial results were published and showed that Rivipansel administered early in VOC results in clinically meaningful benefits for adults and children with SCD, shortening IV opioid use and hospital stay (53).

These efforts by the researchers led to the development of a therapeutic agent targeting P-selectin for the treatment of VOC, which subsequently got approved by the FDA in 2019. The effective anti-adherence agents can act as a blocker to cell–cell interactions that can protect from aggressive erythrocyte aggregation and adherence to vascular endothelium.

One of the newer therapeutic approaches is the induction of fetal hemoglobin (HbF, a2γ2). The HbF is the principal hemoglobin present during the gestation period. Approximately 60%–80% of total hemoglobin in newborns constitutes HbF, it is completely replaced by adult hemoglobin (hemoglobin A, α2β2) at the age of 6–12 months, and in an adult, person HbF constitutes less than 1% of the total hemoglobin. Genome-wide association studies (GWAS) identified BCL11A as a key regulator of hemoglobin switching from HbF to HbA and as a key therapeutic target for HbF induction (54). Pharmacological interventions like the use of Hydroxyurea (HU) resulted in the evaluation of HBF levels in patients with SCD leading to the amelioration of pain crisis (55, 56). Throughout the period, various drugs were tested for induction of HbF, while the most widely used for SCD is HU, and we discussed the mechanism of action of a few of them here. The HU induces HbF by mainly three pathways: (1) epigenetic modification: HU triggers reduced methylation of CpG island, which leads to increased expression of γ-globin. (2) Signal transduction pathway: activation of soluble guanylate cyclase (sGC) leads to the upregulation of the γ-globulin gene at the transcription level in erythroid cell lines and primary erythroblasts (57). (3) Post-transcriptional regulation: HU controls HbF levels by regulating the expression of micro RNAs involved in modulating HbF synthesis. The HU intervention leads to the upregulation of reactive oxygen species (ROS) like NO in patients with SCD, which in turn activates soluble guanylate cyclase (sGC), stimulates cyclic monophosphate (cGMP) and protein kinase G (PKG) pathway for the γ-globulin expression (58). However, various signaling pathways may contribute to the HU-induced NO-cGMP pathway (59). Furthermore, HbF level can also be induced by the combination therapy of HU along with salubrinal, identified as a key regulator of γ-globulin gene expression (60). The drugs like azacytidine and decitabine are used as replacement therapy for HU for HbF induction, mainly for the treatment of HU-resistant patients with SCD; however, later are found potentially more effective in the induction of HbF (61, 62). Other drugs, namely trichostatin-A and valproic acid also observed to play a key role in the induction of HbF (63, 64). Despite the availability of new therapeutic drugs to ameliorate the VOC, none of them proved to be as effective as HU, which can exert an anti-sickling effect through induction of HbF (65). Though, HU has been potentially observed to cure various issues related to SCD e.g. RBC hydration, normalization of neutrophil count, reduction in leukocyte adhesion, and expression of pro-inflammatory molecules, a significant number of patients do not benefit from HU due to suboptimal HbF responses and side effects. Nevertheless, all these efforts well established the HbF as a clinical approach to treating patients with SCD, and the findings led the scientists to consider other approaches like genetics to induce HbF (66). To this end, the identification of the transcriptional regulator of HbF, BCL11A, MYB, and KLF1 completely opened new windows for the treatment of SCD, i.e., intervention through genetic and genomic approaches (67, 68). In 2017, Ribeil and colleagues treated patients with SCD with lentiviral vector-mediated addition of the anti-sickling β globin gene (T87Q) into autologous hematopoietic stem cells (HSCs). The study showed promising results; 15 months after treatment, the level of the anti-sickling β-globin remained high (50%) (69). However, the treatment of choice is HSCT using immunologically matched siblings as donors (70).

**Figure 3:**
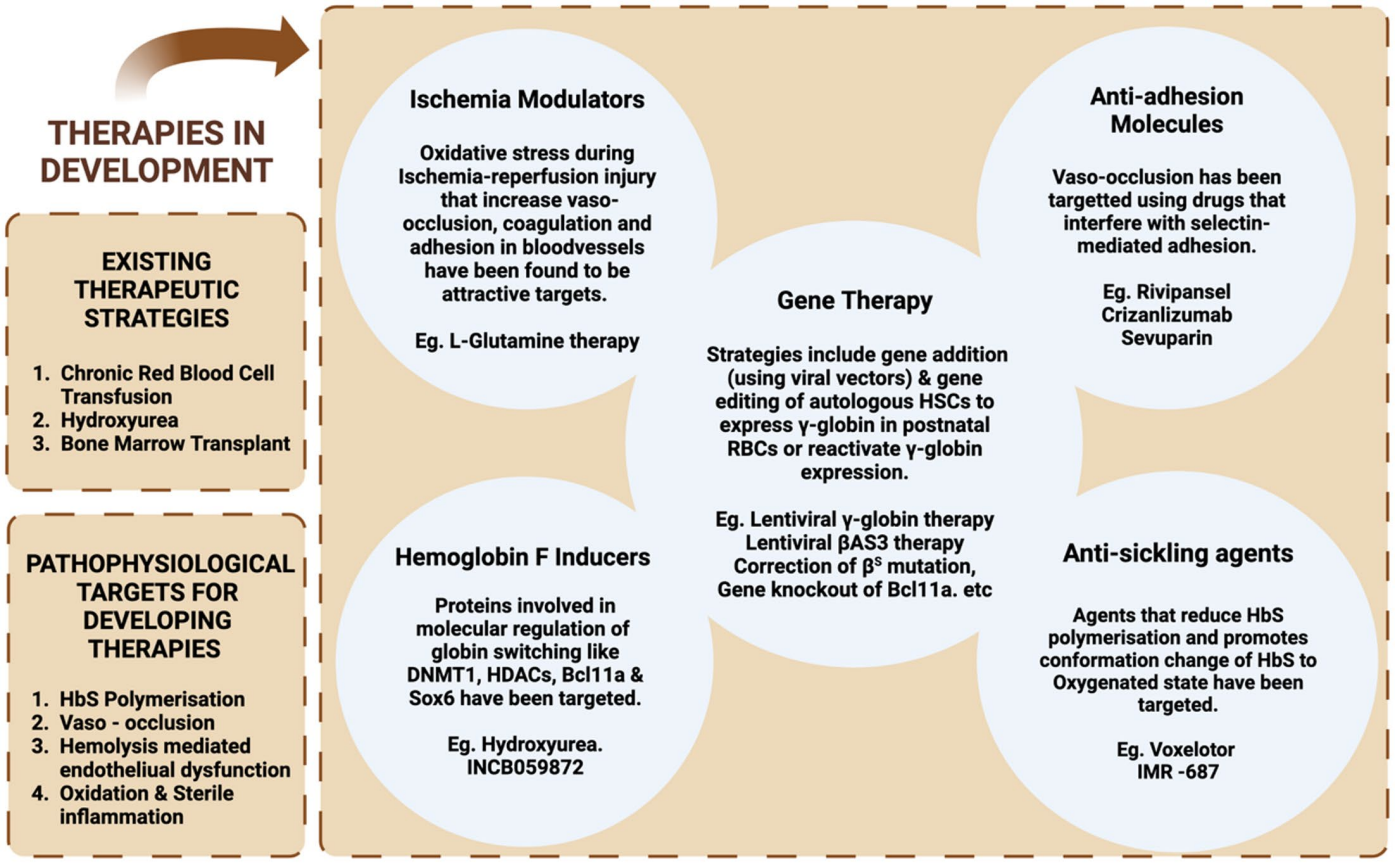
A diagrammatic representation of various therapeutic strategies: current and under development for the treatment of SCD.

Considering the pathophysiology of SCD, different targets can be selected for the introduction of therapeutic interventions. Currently, the major attention has focused on therapeutic approaches for modifying the patient’s genotype. Other targets include the prevention of HbS polymerization, VOC, and inflammation (Figure 3). The HbF induction can overcome the problem of HbS polymerization; however, there are also other approaches to overcome the problem. One approach recently approved by FDA is the increased oxygen affinity of the hemoglobin molecule by Oxbryta (Voxelotor)™ (71). Furthermore, we will discuss in detail about the recent advancements in allogeneic HSCT-based approaches for the treatment of sickle cell disease.

## 5. Allogeneic HSCT-based therapeutic strategies to treat SCD

### 5.1. Matched sibling HSCT under myeloablative conditioning

The first HSCT for SCD was performed in 1984 when an 8-year-old girl with acute myeloid leukemia (AML) and an underlying SCD was subjected to HLA-matched HSCT to cure AML. The patient got cured of leukemia, and at the same time, a reversal in sickle status was observed (72, 73). This pioneer report by Johnson et al. encouraged researchers to go forward with HSCT as a therapeutic option for SCD (73). In 1988, after 4 years of this initial report, Vermylen et al. reported the outcome of matched related donor (MRD) HSCT using myeloablative conditioning with busulfan and cyclophosphamide in five children with SCD. Here, four out of five showed rapid and stable engraftment while one rejected the graft and underwent a second HSCT after 62 days of the first one. Thereafter, among all the children, the outcome of HSCT was uneventful, without any episodes of vaso-occlusion (VOC) and hemolysis with a hemoglobin pattern of donor type (74). Subsequently, in 1996, Walters and colleagues reported the outcome of HLA-matched HSCT in a group of 22 subjects (<16 years), preconditioned with cyclophosphamide, busulfan, and alemtuzumab (ATG). Stable engraftment was observed in 16 patients and stable mixed chimerism was observed in one patient (1/16). Twenty patients survived with a median follow-up of 23.9 months. A total of four patients rejected the graft including one patient with accompanied bone marrow aplasia. The central nervous system hemorrhage or GvHD was the possible cause of death of two patients. The overall survival and event-free survival at 4 years were 91% and 73%, respectively (75).

After 10 years of his first report, Vermylen et al. published the outcome of HLA-matched HSCT under myeloablative conditioning (busulfan, cyclophosphamide, and ATG) in 50 patients. Overall survival, event-free survival, and disease-free survival at 11 years were observed at 93, 82, and 85%, respectively. The study also advocated the advantage of early intervention for a better outcome of HSCT in treating SCD (76). Immediately after this report, Walters et al., in 2000, also reported the outcome of MRD-HSCT with myeloablative conditioning in 50 children with SCD. The patients received matched sibling bone marrow allografts between September 1991 and March 1999, with ages ranging from 3.3 to 15.9 (median 9.9) years. Three patients died due to intracranial hemorrhage or GvHD. Five patients rejected the graft out of 47 surviving patients, five patients rejected the graft with the presence of recurrent SCD, four had stable mixed chimerism, and among 38, full donor chimerism was observed. The authors also reported ovarian dysfunction in five of the seven evaluated female participants (77).

Another report published in 2007 (78) confirmed the findings of Vermylen et al. and Walters et al. The study reported overall survival and event-free survival of 93.1% and 86.1%, respectively. In this study, for the first 12 patients, busulfan and cyclophosphamide combination were used as a myeloablative conditioning regimen. Furthermore, ATG was also included in the regimen to prevent unstable mixed chimerism and rejection with a notably encouraging reduction in rejection rate (22.6% vs. 2.9%). Acute and chronic GvHD was observed in 20% and 12.6% of patients, respectively. The gonadal dysfunction was again observed as a common transplantation-related manifestation (78).

An international survey related to HLA identical sibling HSCT included 1,000 recipients of HLA-identical sibling transplants performed between 1986 and 2013 and reported to the European Society for Blood and Marrow Transplantation, Eurocord, and the Center for International Blood and Marrow Transplant Research. The majority of patients received a myeloablative conditioning regimen (87%) and only 13% of patients received reduced intensity conditioning. The 5-year event-free survival was approximately 91.4% and overall survival was 92.9%. Graft failure was observed in 23 patients and 7% of patients died and the most common cause of transplantation-related death was infection (70).

A very recent study from Spain reported the outcome of myeloablative sibling HSCT in 45 patients. Most patients received a conditioning regimen based on busulfan and cyclophosphamide (69%) and the remaining received treosulfan, thiotepa, and fludarabine. Event-free survival and overall survival at 3 years post-HSCT were 89.4% and 92.1%, respectively. Grade III–IV and chronic GvHD were evident in 6.8% and 5.4% of patients, respectively (79). HSCT studies on patients with SCD involving matched sibling donors are summarized in Supplementary Table 1.

### 5.2. Matched sibling HSCT under non-myeloablative conditioning

Encouraging clinical success was observed with matched sibling myeloablative HSCT; however, associated toxicities with preconditioning regimens like organ damage and gonadal dysfunction make them less attractive to cure SCD especially when patients are already suffering from organ damage due to their SCD status. Moreover, transplantation-related mortality and morbidity can outweigh the morbidity and mortality of SCD. To address these issues, collective effort from clinicians and researchers led to the development of reduced-intensity conditioning (RIC) regimens. HSCT under RIC proved to be advantageous for patients with SCD with underlying organ dysfunction/damage in a safer way which was not possible with myeloablative conditioning regimens. The initial attempts to cure SCD using HSCT under RIC were disappointing. In a study where a non-myeloablative-HSCT approach (fludarabine +200 cGy total body irradiation; two patients also received ATG) was used to treat SCD (six patients) and thalassemia (one patient) followed by a combination of immunosuppressants. Initially, 6 out of 7 patients who underwent HSCT exhibited donor chimerism and patients showed marked correction in hematological parameters. However, once the post-transplantation was immunosuppression tapered, loss of the donor graft, autologous hematopoietic recovery, and disease recurrence was observed (80). A similar outcome was reported in another study where RIC-based HSCT was used to cure a group of 13 patients with non-malignant diseases. Of the four patients with hemoglobinopathies, stable engraftment was observed in only one patient (81). After these initial unsuccessful attempts toward the development of a safer RIC-HSCT approach, the first successful approach was reported by Hsieh et al. in 2009. A total of 10 patients with SCD underwent non-myeloablative conditioning (300 cGy TBI+ alemtuzumab). Sirolimus (rapamycin) was used as GvHD prophylaxis. At a median follow-up of 30 months, stable donor-derived chimerism was observed in nine patients, which was enough to reverse the sickle cell disease status. Interestingly, no evidence of either acute or chronic GvHD was observed (82). In 2014, Hsieh et al. published the outcome of RIC-based sibling HSCT in 30 patients using the same approach. Of 30 patients, 29 patients survived during a median follow-up of 3.4 years, and stable donor chimerism was observed in 26 patients without any evidence of GvHD (83). It also became evident from these studies that alemtuzumab can prevent graft rejection and GvHD by deleting anti-donor host and anti-host donor T cells. Considering the utility of alemtuzumab in RIC-based HSCT for sickle cell disease, other groups also used alemtuzumab-based RIC regimen with reasonably good transplantation outcomes. Bhatia et al. used fludarabine-, busulfan-, and alemtuzumab-based RIC regimen in 18 patients with SCD. A total of 15 and 3 patients received matched sibling bone marrow and cord blood, respectively. All patients showed a higher percentage of donor chimerism. However, grade II-IV GvHD was evident in 17% of patients and chronic GvHD in 11% of patients as well (84). A similar outcome of RIC-based HSCT was reported by King et al. in 2015 (85). In the extended patient number (52 patients: 43 patients with SCD and 8 patients with thalassemia), they demonstrated the utility of RIC-based matched sibling donor HSCT (alemtuzumab, fludarabine, and melphalan). During a three-year follow-up, overall survival and event-free survival were 94.2% and 92.3%, respectively. Acute GvHD and chronic GvHD incidences were observed in 23% and 13% of patients, respectively. Three deaths due to the GvHD were recorded.

Later in 2016 Sharaf et al., confirmed the findings of Hsieh et al., by using RIC-based matched sibling donor HSCT in 13 adult patients with SCD. Stable donor chimerism observed in 12 patients was sufficient to reverse the SCD status without any occurrence of GvHD (86). These findings suggest that 300 cGy TBI is sufficient to create the required niche in the host bone marrow to establish stable donor chimerism. Moreover, alemtuzumab’s lymph-toxic effect persists for weeks that deletes both host and donor T-cell clones potentially responsible for graft rejection and GvHD.

### 5.3. Matched unrelated and haploidentical donors for HSCT

Though the outcomes of non-myeloablative matched sibling HSCT were encouraging, practically it is impossible to find MSD for every patient with SCD in need of HSCT (75, 77, 82, 87). Thus, the paucity of MSD greatly reduced the HSCT utility to cure SCD. Furthermore, the HSCT experience with SCD also suggests that a reversal in disease patterns could be gained even without the complete replacement of the patient’s stem cells (22, 76, 77, 88, 89). The aforementioned reports potentially suggested that the matched unrelated donor (MUD) or haploidentical donor-based HSCT could be implemented in patients with SCD. In another study, Strocchio et al. (90) reported the outcome of Treosulfan-based myeloablative conditioning in 15 patients with SCD including 6 receiving Matched Unrelated Donor (MUD) HSCT. Here, one out of six patients with MUD-HSCT showed graft failure. Disease-free survival at 5 years among those who received MUD-HSCT was 83%, comparatively lower than those who received MSD-HSCT (96%). However, there was no incidence of GvHD among groups (90). Contrary to this report, Shenoy et al. with a larger cohort of pediatric patients with SCD underwent MUD-HSCT(*n* = 29) using a conditioning regimen of alemtuzumab, fludarabine and melphalan reported only 76% and 69% event-free survival rates at 1 and 2 years, respectively. Notably, there was a higher incidence of GvHD; grade II–IV 28% and chronic 62% (20). Very recently, Gluckman et al. published a report of a retrospective survey on 70 MUD-HSCT in patients with SCD carried out in the European Society for Blood and Marrow Transplantation Centers from 2005 to 2017. The overall survival of transplanted patients was 90% and event-free survival was 76% with the incidence of 25% and 23% of acute and chronic GvHD, respectively (91). These studies witnessed that the MUD-HSCT can expand the available donor pool for patients with SCD but to a limited extent. To this end, Tozatto-Maio et al. in 2020, by estimating the HLA haplotypes of 185 patients and by conducting a search for matched donors in international registries, reported that approximately 47% of patients with SCD had a matched donor (92). Considering the scarcity of donors for patients with SCD, haploidentical HSCTs (haplo-HSCT) were included to increase the donor pool possibly for every patient. The very first report on the use of haploidentical HSCT to cure SCD was published in 2004. A 14-year-old boy received the non-myeloablative conditioning regimen of TBI and fludarabine. Cyclosporine and mycophenolate mofetil were used as GVHD prophylaxis. The graft was rejected with a complete absence of donor cells during the 5-month study (93). Principally, two main approaches were used to overcome the HLA barriers: (1) haplo-HSCT with T-cell-depleted grafts; (2) T-cell replete (TCR) grafts or un-manipulated grafts. Both approaches were increasingly tested and developed to overcome the underlying bidirectional issues of GvHD and graft rejection (94). HSCT studies on patients with SCD involving unmatched donors are summarized in Supplementary Table 2.

#### 5.3.1. Haplo-HSCT using T-cell replete graft

The T-cell replete (TCR) haplo-HSCT is based on *in vivo* strategies to overcome the bidirectional alloreactivity and to induce tolerance across MHC barriers. The use of post-transplantation cyclophosphamide completely revolutionized the field by selective deletion of alloreactive T cells. HSCs are protected from the adverse effect of cyclophosphamide due to the expression of the drug-metabolizing enzyme aldehyde dehydrogenase which is not expressed by T cells (94–97). Moreover, cyclophosphamide-induced preferential expansion of regulatory T cells may also contribute to GvHD prevention (95).

In 2008, Brodsky et al. published the outcome of haplo-HSCT in one patient with SCD. The patient received the conditioning regimen of cyclophosphamide, TBI, and fludarabine, and the GvHD prophylaxis regimen of tacrolimus, mycophenolate mofetil along with post-transplantation cyclophosphamide. The patients with SCD exhibited donor-type chimerism and during a follow-up of the 1-year patient remained in remission without any evidence of GvHD (98). In 2012, Bolanos-Meade and colleagues explored the use of haplo-HSCT and post-transplantation cyclophosphamide in an extended number of patients (*n* = 17). Of the 17 patients, 14 patients transplanted with allografts from haploidentical-related donors were preconditioned with ATG, fludarabine, TBI, and cyclophosphamide. Mycophenolate mofetil and post-transplantation cyclophosphamide (days +3 and +4) were used as GvHD prophylaxis. During a median follow-up of 711 days, donor cell engraftment was observed in only 57% of patients with SCD, while the remaining 43% rejected the graft. Autologous hematopoiesis recovery was observed in all patients who rejected the graft. GvHD was not observed in any of the transplanted patients and overall survival was 100% (19). In this study, all the engrafted patients showed correction in SCD status. Moreover, the study also highlighted the need for improvement in the current TCR haplo-HSCT and post-transplantation cyclophosphamide approach.

To address the issues of GvHD and graft failure, Fitzhugh et al. introduced a modified non-myeloablative conditioning regimen with ATG, 400 cGy TBI, and escalating doses of post-transplantation cyclophosphamide (0, 50, and 100 mg/kg) for haplo-HSCT. A total of 23 patients (21 patients with SCD and 2 patients with thalassemia) were included in the study. The patients were distributed in three cohorts based on escalating doses of cyclophosphamide. The patients of cohort 1 (*n* = 3) did not receive post-transplantation cyclophosphamide, whereas those in cohort 2 (*n* = 8) and cohort 3 (*n* = 12) received 50 and 100 mg/kg cyclophosphamide, respectively. The results showed that 10 out of 12 patients from cohort 3 developed donor chimerism, whereas only five out of eight and one out of three patients showed engraftment from cohorts 1 and 2, respectively. After 1 year of post-transplantation, 50% of patients from cohort 3 remained disease free which was the highest among the groups. Moreover, the incidence of GvHD was observed in all cohorts of patients (no GvHD in cohort 1, grade I acute GvHD in one patient from each cohort 2 and 3) (99). In this study, the conditioning protocol modifications weighing to minimize the occurrence of GvHD was possibly the major cause of rejections resulted. However, in cohort 3, stable engraftment was observed in six (50%) of transplanted subjects who received 100 mg/kg post-transplantation cyclophosphamide. Though the outcomes were not very promising, this study set a platform for the future use of post-transplantation cyclophosphamide.

Saraf et al. reported the outcome of haplo-HSCT in 10 patients. The first two patients received the conditioning regimen of alemtuzumab, 300 cGy TBI, and cyclophosphamide of 50 mg/kg on days +3 and 4. Both the patients rejected the graft, so they adopted John Hopkins university’s conditioning protocol. For the remaining eight patients, they used mobilized blood as a source of stem cells and a conditioning regimen containing TBI 300 cGy, ATG, cyclophosphamide, and fludarabine. Post-transplantation cyclophosphamide (days +3 and 4; 50 mg/kg) along with mycophenolate mofetil and sirolimus was used as GvHD prophylaxis. The engraftment was observed in all eight patients; secondary graft failure was observed in one patient at day 90 who showed autologous hematopoietic recovery. Two patients developed acute GvHD and one chronic patient developed chronic GvHD and later died due to unexplained reasons. With a median follow-up of 16 months, the remaining seven patients were alive. The study highlighted the safety of the modified John Hopkins approach (86). Although higher engraftment and reduced mortality were observed using the modified John Hopkins approach, this approach was not devoid of transplantation-related toxicities and viral reactivation.

At the same time, another multicentric study published a report of 16 patients with SCD who underwent haplo-HSCT under non-myeloablative conditioning and post-transplantation cyclophosphamide (John Hopkins approach) (100). For the first three patients conditioning regimen was ATG, fludarabine, cyclophosphamide, and 200 cGy TBI; GvHD prophylaxis consisted of post-transplantation cyclophosphamide, mycophenolate mofetil, and sirolimus. Two out of three patients showed primary graft failure. To overcome the problem of graft rejection, thiotepa was included in the conditioning regimen and then the remaining 13 and 2 patients who previously rejected the graft underwent haplo-HSCT with thiotepa augmented conditioning regimen. During a median follow-up of 13.3 months, 14 of 15 patients had donor engraftment > 95% with 100% overall survival. Grades III–IV GVHD occurred in only one patient and chronic GvHD was also observed in only one patient. This study suggested that the use of post-transplantation cyclophosphamide and thiotepa in a conditioning regimen (John Hopkins approach) improves donor engraftment without the higher incidence of fatal GvHD. However, again there was an incidence of viral reactivation and transplantation-related toxicities among transplanted patients.

A second study by Bolanos-Meade et al. on 12 patients with SCD reported the outcome of haplo-HSCT with a modified regimen (400 cGy TBI, ATG, fludarabine, cyclophosphamide, and post-transplantation cyclophosphamide) (101). Graft failure was observed in one patient (8%), and overall survival was 100%. Three patients (25%) experienced acute GVHD, and one patient developed chronic GVHD.

Kharya et al. (102) reported the outcome of haplo-HSCT in 25 patients using mobilized blood graft instead of bone marrow. All the patients received the conditioning regimen used by de la Fuente et al., in 2019 (100). Two courses of immune suppression were used to prevent GvHD, pre-transplant (fludarabine, cyclophosphamide, and dexamethasone), and post-transplant (cyclophosphamide, sirolimus, and mycophenolate mofetil). Engraftment was observed in all the patients. Unfortunately, 12% (3) died due to transplantation-related mortality. Overall survival was 88% with the incidence of acute and chronic GvHD in 20% and 12% of patients, respectively.

#### 5.3.2. Haplo-HSCT using T-cell-depleted graft

Graft-derived T cells are considered key players in the occurrence of GvHD. *Ex vivo* depletion of T cells from the graft is one of the effective approaches for the prevention of GvHD (103). One of the major advantages of this approach is that it restricts the prolonged use of immunosuppressive drugs, which may make the host more prone to infection and can cause multi-organ toxicity (103). The approach involves either extensive depletion of T cells from hematopoietic grafts or a positive selection of CD34+ cells. Later it can cause a delay in immune reconstitution due to the positive selection of CD34 graft lacking B and NK cells that do not happen during T-cell depletion.

Initial attempts to deplete T cells from hematopoietic grafts were carried out in the late 1980s *via* agglutination with soybean lectin and resetting the residual T cells with sheep RBC (104–106), and the approach was further advanced to use the monoclonal antibodies directed against T cells (106, 107). Initially, the approach was observed as potentially useful in the prevention of GvHD with increased cases of relapse among patients with leukemia (108). Moreover, graft failure was another issue associated with the TCD approach (109). These findings suggested that donor T cells have a promising role in graft survival; they can prevent graft rejection by counteracting against residual host immune cells left after preconditioning. Despite this, encouraging results have been observed by using TCD hematopoietic graft.

Dallas et al. (110) reported the outcome of TCD-haplo-HSCT in eight SCD children with a median age of 9.0 ± 5.0. The CD34+ cells were selected by CliniMACS (Miltenyi Biotech) and *in vivo* CD3 depletion was accompanied by muromonab. During a median follow-up of 7.4 ± 2.4 years, overall survival and disease-free survival were observed at 75% and 38%, respectively. The incidence of acute and chronic GvHD was 50 and 38%, respectively. The major aim of the study was to expand the donor pool availability with a reduced rate of GvHD and sustainable long-term outcomes of HSCT.

Foell et al. (21) published the report of haplo-HSCT in nine patients who failed hydroxyurea treatment, by using a Treosulfan-based conditioning regimen using T-and B-cell-depleted haploidentical grafts from first-degree related relatives who were fully haplotype mismatched. The myeloablative conditioning regimen consisted of ATG, fludarabine, and treosulfan. Considering the higher chances of graft rejection, immunosuppression was continued up to 120 days post-HSCT by using cyclosporine and mycophenolate mofetil. Stable donor cell engraftment was observed in all patients during a median follow-up of 26 months. The study aimed to test whether treosulfan is effective in creating sufficient niches for HSCT with reduced toxicities and GvHD. Despite the myeloablative nature of conditioning, it was tolerable among the patients, and only mild grade 1–2 toxicities were observed. However, viral reactivation emerged as a common problem. However, this terosulfan-based novel conditioning regimen opened a new door for HSCT to induce engraftment with reduced transplantation toxicities. The same year, Gilman et al. (111) reported the outcome of haplo-HSCT by using a RIC regimen and CD34+ selected or T-cell-depleted graft in 10 patients. The conditioning regimen consisted of melphalan, thiotepa, fludarabine, and ATG. Methotrexate was used as GvHD prophylaxis. During a median follow-up of 49 months, 9 out of 10 survived with stable donor engraftment and without sickle cell disease complications. Acute and chronic GvHD was observed in two and one patients, respectively. The viral reactivation again emerged as an issue that needed to be addressed.

Very recently, Gaziev and colleagues showed the results of haplo-HSCT by using T-cell receptor αβ+/CD19+ depleted grafts (112). The conditioning regimen consisted of busulfan, thiotepa, cyclophosphamide, and ATG preceded by fludarabine, hydroxyurea, and azathioprine. A total of 14 patients, 11 with thalassemia and 3 with SCD, were included in the study. The resultant data were compared with the outcome of haplo-HSCT among a group of 40 patients by using CD34+ selected from peripheral blood and bone marrow (*n* = 32), CD34+ selected from peripheral blood, and CD3+/CD19+ from depleted bone marrow grafts (*n* = 8). The 5-year probability of overall survival and disease-free survival were observed at 84% and 69%, respectively. The incidence of graft failure was 14%. The results showed that a higher incidence of graft failure was observed in the CD34 group (45%) when compared with the TCR group (14%). The incidence of acute and chronic GvHD was comparable among the groups (28% vs. 29% and 10% vs. 21%, respectively). Viral reactivation was also common in both groups. The study showed that TCR αβ+/CD19+ depleted grafts were associated with reduced incidence of GvHD as well as a delay in immune reconstitution associated with mortality and morbidity remained a challenge to be addressed (112).

Similarly, the occurrence of GvHD and graft failure was observed in a very recent study that reported the outcome of 25 patients with SCD who underwent haplo-HSCT using a myeloablative conditioning regimen (ATG, fludarabine, and treosulfan). The graft was T and B cell depleted. Secondary graft failure was observed in two patients who died due to transplantation-related complications. Another patient died due to viral reactivation reducing the overall survival to 88%. The incidence of acute GvHD was 32% (7 out of 25), and 18% (4 out of 25) developed mild to moderate GvHD (113). The patients in the MSD-SCT group had an OS of 100%, and no graft failure occurred. The incidence of grade I-II acute GVHD was 23%, and mild to moderate chronic GvHD occurred in two patients (15%).

#### 5.3.3. Veto cells-based TCD approach

**Figure 4:**
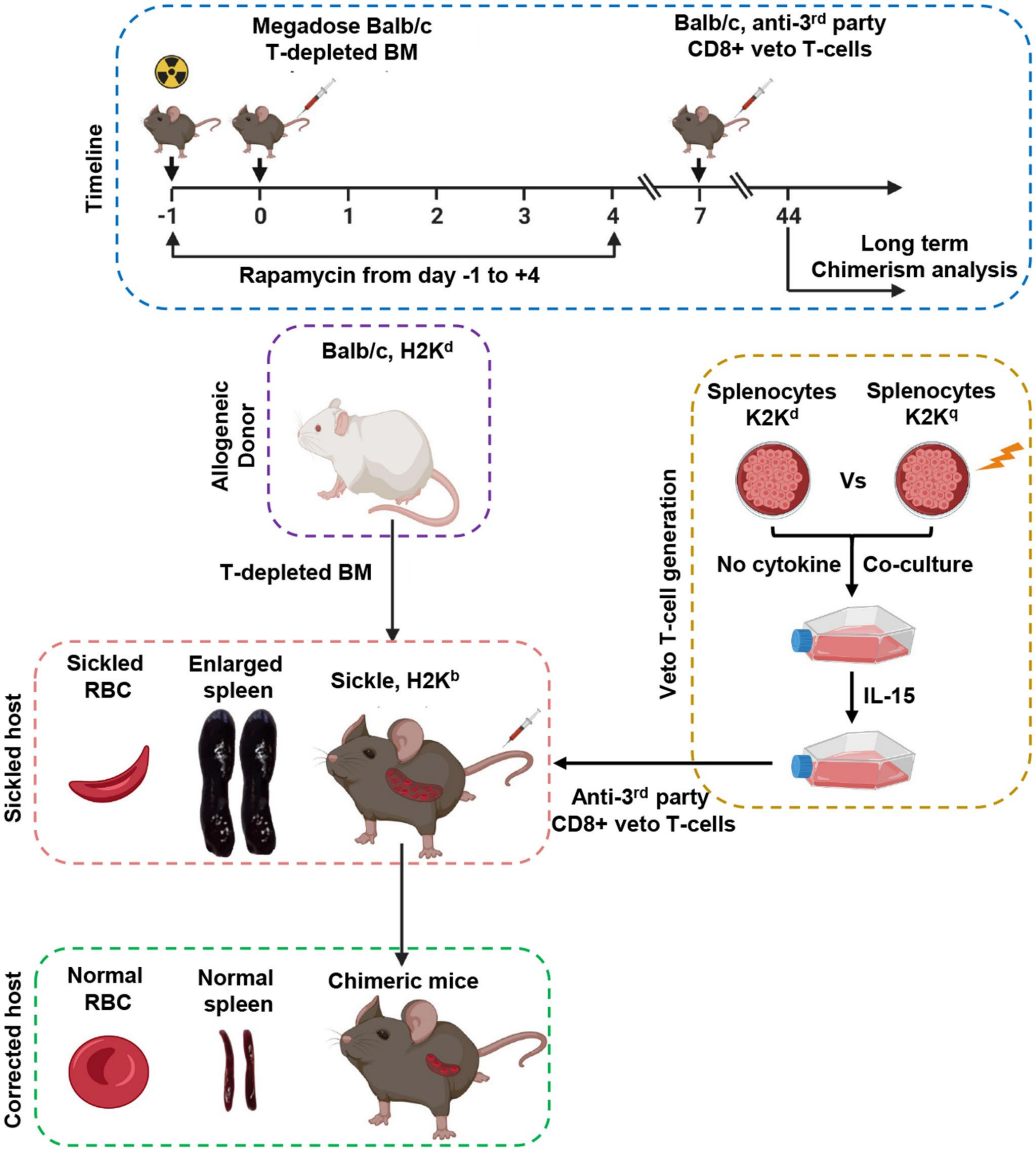
Schematic representation of the allogeneic hematopoietic stem transplantation with anti-third party veto cells under RIC to correct sickle cell disease in a murine model.

Both the approaches either TCR or TCD showed encouraging success in the treatment of SCD by increasing donor availability, improving engraftment, and minimizing the risk of GvHD. However, both tested approaches with different conditioning regimens failed to establish the ideal protocol for HSCT to cure SCD without graft failure, the incidence of GvHD, and viral reactivation. To this end, a preclinical study by Singh and colleagues in a well-defined SCD mice model showed that durable engraftment of allogeneic hematopoietic stem cells could be achieved by using donor-derived CD8+ veto T cells with a complete reversal in disease pattern (Figure 4). The authors reported that graft rejection of T-cell-depleted non-myeloablative HSCT can be overcome in fully mismatched SCD recipient mice by using a mega dose of bone marrow, anti-third party veto cells, and short-term dose of rapamycin without the occurrence of GvHD. The current approach is under phase I/II clinical trial to cure hematological malignancies (NCT03622788). The approach is based on the generation of antiviral donor-derived CD8+ veto T cells. Extensive research by Prof. Yair Reisner’s group provided sufficient information that anti-third party CD8+ veto can induce tolerance and engraftment of haplo-HSCs by specifically deleting only alloreactive T cells directed against veto cells thus sparing non-alloreactive immune cells to fight against infection (114, 115). The veto cells directed against viral antigens do not cause GvHD and would also protect against viral reactivation a common problem in transplantation settings. The clinical trial results are awaited, their success will open a new door to treating hematological or non-hematological disease by HSCT without the risk of graft failure, GvHD, and viral reactivation under reduced intensity conditioning by using megadose of peripheral blood stem cells and donor derive CD8+ veto T cells.

## 6. Conclusion

Tremendous success has been achieved by MSD and haplo-HSCT to cure SCD. However, in every approach, the incidence of GvHD, graft failure, and viral reactivation are existing issues. To this end, Veto CD8 T-cell-based haplo-HSCT approach seems promising in overcoming HLA barriers as well as viral reactivation. Only preclinical data are currently available right now. A clinical trial testing the safety and efficacy of central memory CD8 veto cells in recipients of non-myeloablative T-cell-depleted HLA-haplotype-matched transplants in patients with hematological cancers is in progress. Furthermore, different gene therapy-based approaches are also underway in clinical trials showing promising results. However, all of these approaches are in their early phase still a long road to go and allo-HSCT is the only treatment of choice for the treatment of SCD.

## Author contributions

AS and SKY conceptualized, supervised, and co-wrote the manuscript. NB and AB facilitated a compilation of relevant literature, manuscript writing, figure preparation, and participated in discussions. All authors contributed to the article and approved the submitted version.

## Conflict of interest

The authors declare that the research was conducted in the absence of any commercial or financial relationships that could be construed as a potential conflict of interest.

## Publisher’s note

All claims expressed in this article are solely those of the authors and do not necessarily represent those of their affiliated organizations, or those of the publisher, the editors and the reviewers. Any product that may be evaluated in this article, or claim that may be made by its manufacturer, is not guaranteed or endorsed by the publisher.
