# Supplementary material for: Allogeneic hematopoietic stem cell transplantation to cure sickle cell disease: A review
Source: Front Med (Lausanne). 2023 Feb 23;10:1036939. doi: 10.3389/fmed.2023.1036939 (PMC9995916; doi:10.3389/fmed.2023.1036939)
Supplement: Supplementary file 1 [file Table_1.DOCX]

Table 1: A summary of studies on matched donor HSCT for SCD.

| **S. No.** | **N** | **Median Follow up** | **Stem Cell Source** | **Conditioning Type** | **Conditioning Regimen** | **TRM** | **OS** | **GF** | **GvHD** | | **References** |
| --- | --- | --- | --- | --- | --- | --- | --- | --- | --- | --- | --- |
|  |  |  |  |  |  |  |  |  | **Acute** | **Chronic** |  |
| 1. | 5 | 10 months (3-21) | BM | MA | Bu and Cyclo |  | 100% | 1 | 40% | NR | [1] |
| 2. | 22 | 23.9 months (10.1-51.0) | BM | MA | Cyclo, Bu, ALZ or ATG. |  | 91% | 3 | 9% | NR | [2] |
| 3. | 50 | 57.9 months (38-95) | BM | MA | Bu + Cyclo and ATG  or CAMPATH IG |  | 94% | 5 | NR | 6% | [3] |
| 4. | 12 | 15 Years (2-61) Years | BM | MA | Bu and Cyclo +ATG |  | 93.1% |  | 20% | 12.6% | [4] |
| 5. | 1000 | 54.5 (0.3-324.6) months | BM (84%), PBSC (7%), CB (9%) | MA | Bu +Cyclo (69%) and Treo + Thio +Flu |  | 92.9% | 23 | 14.8% | 14.3% | [5] |
| 6. | 45 | <5 years: 24 month (10 – 133)  >5 years: 41 (11 – 246) | BM | MA | Bu+ Cyclo |  | 92.1 % | 0 | 6.8% | 5.4% | [6] |
| 7. | 7;  SCD (6), β-Th (1) | 28 months (16–40) | BM (6), PBSC (1) | NMA | Flu+200cGy TBI, ATG and MMF + CYSP or Tac |  |  | 7 | 14.2% | NR | [7] |
| 8 | 10 | 30 months (15-54) | CD34+ PBSC | NMA | 300 cGy TBI, ALZ + Sirolimus |  | 100% | 1 | 0 | 0 | [8] |
| 9 | 30 | 3.4 years (1–8.6) |  | NMA | ALZ, 300 cGy TBI, Sirolimus |  | 96.6% |  | NR | NR | [9] |
| 10 | 18 | 1065.5 days (135–2731) | BM (15),  CB (3) | MA | Flu, Bu and ALZ |  | 100% |  | 17% | 11% | [10] |
| 11 | 52; SCD (43), β-Th (9) | 3.42 years  (0.75–11.83) | BM (46), CB (5), BM+CB (1) | MA | ALZ, Flu, and MLP |  | SCD (94%); β-Th (100%) |  | 23% | 13% | [11] |
| 12 | 13 | 22 months (12 to 44) | PBSC | NMA | ALZ/300 cGy TBI, Sirolimus |  | 100% | 1 | 0 | 0 | [12] |

Number of Patient (N), Overall Survival (OS), Number of patient experienced Graft Failure (GF), Transplantation Related Mortality (TRM), Graft versus Host Disease (GvHD), Not reported (NR), Bone marrow (BM), Cord blood cells (CB), Peripheral Blood Stem Cell (PBSC), Myeloablative (MA), Non- myeloablative (NMA), Fludarabine (Flu), Cyclophosphamide (Cyclo), Busulfan (Bu), Alemtuzumab (ALZ), Anti-Thymocyte Globulin (ATG), Treosulfan (Treo), Thiotepa (Thio), Mycophenolate Mofetil (MMF), Cyclosporine (CYSP), Melphalan (MLP), Hydroxyurea (HU), Azathioprine (AZA).

**References**

[1] C. Vermylen, E. Fernandez Robles, J. Ninane, and G. Cornu, Bone marrow transplantation in five children with sickle cell anaemia. Lancet 1 (1988) 1427-8.

[2] M.C. Walters, M. Patience, W. Leisenring, J.R. Eckman, G.R. Buchanan, Z.R. Rogers, N.E. Olivieri, E. Vichinsky, S.C. Davies, W.C. Mentzer, D. Powars, J.P. Scott, F. Bernaudin, K. Ohene-Frempong, P.J. Darbyshire, A. Wayne, I.A. Roberts, P. Dinndorf, S. Brandalise, J.E. Sanders, D.C. Matthews, F.R. Appelbaum, R. Storb, and K.M. Sullivan, Barriers to bone marrow transplantation for sickle cell anemia. Biol Blood Marrow Transplant 2 (1996) 100-4.

[3] M.C. Walters, R. Storb, M. Patience, W. Leisenring, T. Taylor, J.E. Sanders, G.E. Buchanan, Z.R. Rogers, P. Dinndorf, S.C. Davies, I.A. Roberts, R. Dickerhoff, A.M. Yeager, L. Hsu, J. Kurtzberg, K. Ohene-Frempong, N. Bunin, F. Bernaudin, W.Y. Wong, J.P. Scott, D. Margolis, E. Vichinsky, D.A. Wall, A.S. Wayne, C. Pegelow, R. Redding-Lallinger, J. Wiley, M. Klemperer, W.C. Mentzer, F.O. Smith, and K.M. Sullivan, Impact of bone marrow transplantation for symptomatic sickle cell disease: an interim report. Multicenter investigation of bone marrow transplantation for sickle cell disease. Blood 95 (2000) 1918-24.

[4] F. Bernaudin, G. Socie, M. Kuentz, S. Chevret, M. Duval, Y. Bertrand, J.P. Vannier, K. Yakouben, I. Thuret, P. Bordigoni, A. Fischer, P. Lutz, J.L. Stephan, N. Dhedin, E. Plouvier, G. Margueritte, D. Bories, S. Verlhac, H. Esperou, L. Coic, J.P. Vernant, E. Gluckman, and T.C. Sfgm, Long-term results of related myeloablative stem-cell transplantation to cure sickle cell disease. Blood 110 (2007) 2749-56.

[5] E. Gluckman, B. Cappelli, F. Bernaudin, M. Labopin, F. Volt, J. Carreras, B. Pinto Simoes, A. Ferster, S. Dupont, J. de la Fuente, J.H. Dalle, M. Zecca, M.C. Walters, L. Krishnamurti, M. Bhatia, K. Leung, G. Yanik, J. Kurtzberg, N. Dhedin, M. Kuentz, G. Michel, J. Apperley, P. Lutz, B. Neven, Y. Bertrand, J.P. Vannier, M. Ayas, M. Cavazzana, S. Matthes-Martin, V. Rocha, H. Elayoubi, C. Kenzey, P. Bader, F. Locatelli, A. Ruggeri, M. Eapen, t.P.W.P.o.t.E.S.f.B. Eurocord, T. Marrow, B. the Center for International, and R. Marrow Transplant, Sickle cell disease: an international survey of results of HLA-identical sibling hematopoietic stem cell transplantation. Blood 129 (2017) 1548-1556.

[6] M.I. Benitez-Carabante, C. Belendez, M. Gonzalez-Vicent, L. Alonso, M.L. Uria-Oficialdegui, M. Torrent, J.M. Perez-Hurtado, J.L. Fuster, E. Cela, C. Diaz-de-Heredia, and G.E.d.T.H. Grupo Espanol de Trasplante de Medula Osea en Ninos, Matched sibling donor stem cell transplantation for sickle cell disease: Results from the Spanish group for bone marrow transplantation in children. Eur J Haematol 106 (2021) 408-416.

[7] R. Iannone, J.F. Casella, E.J. Fuchs, A.R. Chen, R.J. Jones, A. Woolfrey, M. Amylon, K.M. Sullivan, R.F. Storb, and M.C. Walters, Results of minimally toxic nonmyeloablative transplantation in patients with sickle cell anemia and beta-thalassemia. Biol Blood Marrow Transplant 9 (2003) 519-28.

[8] M.M. Hsieh, E.M. Kang, C.D. Fitzhugh, M.B. Link, C.D. Bolan, R. Kurlander, R.W. Childs, G.P. Rodgers, J.D. Powell, and J.F. Tisdale, Allogeneic hematopoietic stem-cell transplantation for sickle cell disease. N Engl J Med 361 (2009) 2309-17.

[9] M.M. Hsieh, C.D. Fitzhugh, R.P. Weitzel, M.E. Link, W.A. Coles, X. Zhao, G.P. Rodgers, J.D. Powell, and J.F. Tisdale, Nonmyeloablative HLA-matched sibling allogeneic hematopoietic stem cell transplantation for severe sickle cell phenotype. JAMA 312 (2014) 48-56.

[10] M. Bhatia, Z. Jin, C. Baker, M.B. Geyer, K. Radhakrishnan, E. Morris, P. Satwani, D. George, J. Garvin, G. Del Toro, W. Zuckerman, M.T. Lee, M. Licursi, R. Hawks, E. Smilow, L.A. Baxter-Lowe, J. Schwartz, and M.S. Cairo, Reduced toxicity, myeloablative conditioning with BU, fludarabine, alemtuzumab and SCT from sibling donors in children with sickle cell disease. Bone Marrow Transplant 49 (2014) 913-20.

[11] A.A. King, N. Kamani, N. Bunin, I. Sahdev, J. Brochstein, R.J. Hayashi, M. Grimley, A. Abraham, J. Dioguardi, K.W. Chan, D. Douglas, R. Adams, M. Andreansky, E. Anderson, A. Gilman, S. Chaudhury, L. Yu, J. Dalal, G. Hale, G. Cuvelier, A. Jain, J. Krajewski, A. Gillio, K.A. Kasow, D. Delgado, E. Hanson, L. Murray, and S. Shenoy, Successful matched sibling donor marrow transplantation following reduced intensity conditioning in children with hemoglobinopathies. Am J Hematol 90 (2015) 1093-8.

[12] S.L. Saraf, A.L. Oh, P.R. Patel, Y. Jalundhwala, K. Sweiss, M. Koshy, S. Campbell-Lee, M. Gowhari, J. Hassan, D. Peace, J.G. Quigley, I. Khan, R.E. Molokie, L.L. Hsu, N. Mahmud, D.J. Levinson, A.S. Pickard, J.G. Garcia, V.R. Gordeuk, and D. Rondelli, Nonmyeloablative Stem Cell Transplantation with Alemtuzumab/Low-Dose Irradiation to Cure and Improve the Quality of Life of Adults with Sickle Cell Disease. Biol Blood Marrow Transplant 22 (2016) 441-8.
