# Supplementary material for: Allogeneic hematopoietic stem cell transplantation to cure sickle cell disease: A review
Source: Front Med (Lausanne). 2023 Feb 23;10:1036939. doi: 10.3389/fmed.2023.1036939 (PMC9995916; doi:10.3389/fmed.2023.1036939)
Supplement: Supplementary file 2 [file Table_2.DOCX]

Table 2: A summary of studies on unmatched donor HSCT for SCD based on T-cell replete or T-cell deplete graft.

| **S. No.** | **N** | **Median Follow up** | **Stem Cell Source** | **Conditioning Type** | **Conditioning Regimen** | **Graft Type** | **TRM** | **OS** | **GF** | **GvHD** | | **References** |
| --- | --- | --- | --- | --- | --- | --- | --- | --- | --- | --- | --- | --- |
|  |  |  |  |  |  | **TCR/TCD** |  |  |  | **Acute** | **Chronic** |  |
| 1. | 23; SCD (21), β-Tha (2) | 3.17 years (0.67- 6.16) | PBSC | NMA | ALZ, 400 cGy TBI, Sirolimus and PTCy | TCD |  | 87% | 1 | 9.5% | 4% | [1] |
| 2. | 15 | 6·3 years (0·3–16·2) | BM, CB, PBSC | MA | Treo, Thio, Flu | TCD |  | 100% | 2 | 7% | NR | [2] |
| 3. | 52 | 26 months (12-62) | BM | MA | ALZ, Flu and MLP | TCD |  | 84.6% | 6 | 23% | 13% | [3] |
| 4. | 71 | 38 months (2–154) | BM, PBSC | MA | Flu, Thio + Treo (64%) or Bu + Cyclo (12%). | TCD |  | 90% | 7 | 25% | 23% | [4] |
| 5. | 17 | 22.9 months (7.2-63.9) | BM | NMA | ATG, Flu, Cyclo, 2 Gy TBI, PTCy | TCR |  | 100% | 7 | 0 | 0 | [5] |
| 6. | 21 | 37.3 months (7.9-72.5) | PBSC | NMA | ALZ, 4 Gy TBI, PTCy | TCR |  | 86% | 12 | 9% | 4% | [1] |
| 7. | 8 | 16.4 months (11.7 - 30) | PBSC | NMA | ATG, Flu, Cyclo, 3 Gy TBI, PTCy | TCR |  | 87% | 10 | 25% | 13% | [6] |
| 8. | 16 | 13.3 months (3.8- 23.1) | BM | NMA | ATG, Flu, Cyclo, and 200 cGy TBI, PTCy | TCR |  | 100% | 3 | 33% | 7% | [7] |
| 9. | 9 | 26 months | PBSC | MA | ATG, Flu, Thio, Treo, CD3+/CD19+ depleted | TCD | 11 | 89% | 0 | 56% | 11% | [8] |
| 10. | 10 | 49 months (14-60) | PBSC | MA | MLP, Thio, Flu, ATG | TCD |  | 90% | 1 | 20% | 10% | [9] |
| 11. | 14; SCD (3), β-Tha (11) | 3.9 years | BM, PBSC | MA | Bu, Thio, Cyclo, ATG, Flu, HU, AZA | TCR |  | 84% | 2 | 28% | 21% | [10] |

Number of Patient (N), Overall Survival (OS), Number of patient experienced Graft Failure (GF), Transplantation Related Mortality (TRM), Graft versus Host Disease (GvHD), T- cell repletion (TCR), T-cell Depletion (TCD), Not reported (NR), Bone marrow (BM), Cord blood cells (CB), Peripheral blood stem cell (PBSC), Myeloablative (MA), Non- myeloablative (NMA), Fludarabine (Flu), Cyclophosphamide (Cyclo), Post-transplantation Cyclophosphamide (PTCy), Busulfan (Bu), Alemtuzumab (ALZ), Anti-Thymocyte Globulin (ATG), Treosulfan (Treo), Thiotepa (Thio), Mycophenolate Mofetil (MMF), Cyclosporine (CYSP), Melphalan (MLP), Hydroxyurea (HU), Azathioprine (AZA).

**References**

[1] C.D. Fitzhugh, M.M. Hsieh, T. Taylor, W. Coles, K. Roskom, D. Wilson, E. Wright, N. Jeffries, C.J. Gamper, J. Powell, L. Luznik, and J.F. Tisdale, Cyclophosphamide improves engraftment in patients with SCD and severe organ damage who undergo haploidentical PBSCT. Blood Adv 1 (2017) 652-661.

[2] L. Strocchio, M. Zecca, P. Comoli, T. Mina, G. Giorgiani, E. Giraldi, L. Vinti, P. Merli, M. Regazzi, and F. Locatelli, Treosulfan-based conditioning regimen for allogeneic haematopoietic stem cell transplantation in children with sickle cell disease. Br J Haematol 169 (2015) 726-36.

[3] S. Shenoy, M. Eapen, J.A. Panepinto, B.R. Logan, J. Wu, A. Abraham, J. Brochstein, S. Chaudhury, K. Godder, A.E. Haight, K.A. Kasow, K. Leung, M. Andreansky, M. Bhatia, J. Dalal, H. Haines, J. Jaroscak, H.M. Lazarus, J.E. Levine, L. Krishnamurti, D. Margolis, G.C. Megason, L.C. Yu, M.A. Pulsipher, I. Gersten, N. DiFronzo, M.M. Horowitz, M.C. Walters, and N. Kamani, A trial of unrelated donor marrow transplantation for children with severe sickle cell disease. Blood 128 (2016) 2561-2567.

[4] E. Gluckman, J. Fuente, B. Cappelli, G.M. Scigliuolo, F. Volt, K. Tozatto-Maio, V. Rocha, M. Tommaso, F. O'Boyle, F. Smiers, C.B.D. Cunha-Riehm, E. Calore, S. Bonanomi, S. Graphakos, A. Paisiou, M.H. Albert, A. Ruggeri, M. Zecca, A.C. Lankester, S. Corbacioglu, D. Paediatric, and E. Inborn Errors Working Parties of the, The role of HLA matching in unrelated donor hematopoietic stem cell transplantation for sickle cell disease in Europe. Bone Marrow Transplant 55 (2020) 1946-1954.

[5] J. Bolanos-Meade, E.J. Fuchs, L. Luznik, S.M. Lanzkron, C.J. Gamper, R.J. Jones, and R.A. Brodsky, HLA-haploidentical bone marrow transplantation with posttransplant cyclophosphamide expands the donor pool for patients with sickle cell disease. Blood 120 (2012) 4285-91.

[6] S.L. Saraf, A.L. Oh, P.R. Patel, K. Sweiss, M. Koshy, S. Campbell-Lee, M. Gowhari, S. Jain, D. Peace, J.G. Quigley, I. Khan, R.E. Molokie, N. Mahmud, V.R. Gordeuk, and D. Rondelli, Haploidentical Peripheral Blood Stem Cell Transplantation Demonstrates Stable Engraftment in Adults with Sickle Cell Disease. Biol Blood Marrow Transplant 24 (2018) 1759-1765.

[7] J. de la Fuente, N. Dhedin, T. Koyama, F. Bernaudin, M. Kuentz, L. Karnik, G. Socie, K.A. Culos, R.A. Brodsky, M.R. DeBaun, and A.A. Kassim, Haploidentical Bone Marrow Transplantation with Post-Transplantation Cyclophosphamide Plus Thiotepa Improves Donor Engraftment in Patients with Sickle Cell Anemia: Results of an International Learning Collaborative. Biol Blood Marrow Transplant 25 (2019) 1197-1209.

[8] J. Foell, B. Pfirstinger, K. Rehe, D. Wolff, E. Holler, and S. Corbacioglu, Haploidentical stem cell transplantation with CD3(+)-/CD19(+)- depleted peripheral stem cells for patients with advanced stage sickle cell disease and no alternative donor: results of a pilot study. Bone Marrow Transplant 52 (2017) 938-940.

[9] A.L. Gilman, M.J. Eckrich, S. Epstein, C. Barnhart, M. Cannon, T. Fukes, M. Hyland, K. Shah, D. Grochowski, E. Champion, and A. Ivanova, Alternative donor hematopoietic stem cell transplantation for sickle cell disease. Blood Adv 1 (2017) 1215-1223.

[10] J. Gaziev, A. Isgro, P. Sodani, K. Paciaroni, G. De Angelis, M. Marziali, M. Ribersani, C. Alfieri, A. Lanti, T. Galluccio, G. Adorno, and M. Andreani, Haploidentical HSCT for hemoglobinopathies: improved outcomes with TCRalphabeta(+)/CD19(+)-depleted grafts. Blood Adv 2 (2018) 263-270.
